# Behavioural and Psychiatric Phenotypes in Men and Boys with X-Linked Ichthyosis: Evidence from a Worldwide Online Survey

**DOI:** 10.1371/journal.pone.0164417

**Published:** 2016-10-06

**Authors:** Sohini Chatterjee, Trevor Humby, William Davies

**Affiliations:** 1 MRC Centre for Neuropsychiatric Genetics and Genomics and Division of Psychological Medicine and Clinical Neurosciences, School of Medicine, Cardiff University, Cardiff, United Kingdom; 2 School of Psychology, Cardiff University, Cardiff, United Kingdom; 3 Neuroscience and Mental Health Research Institute, Cardiff University, Cardiff, United Kingdom; TNO, NETHERLANDS

## Abstract

**Background:**

X-linked ichthyosis (XLI) is a rare dermatological condition arising from deficiency for the enzyme steroid sulfatase (STS). Preliminary evidence in boys with XLI, and animal model studies, suggests that individuals lacking STS are at increased risk of developmental disorders and associated traits. However, the behavioural profile of children with XLI is poorly-characterised, and the behavioural profile of adults with XLI has not yet been documented at all.

**Materials and Methods:**

Using an online survey, advertised worldwide, we collected detailed self- or parent-reported information on behaviour in adult (n = 58) and younger (≤18yrs, n = 24) males with XLI for comparison to data from their non-affected brothers, and age/gender-matched previously-published normative data. The survey comprised demographic and background information (including any prior clinical diagnoses) and validated questionnaires assaying phenotypes of particular interest (Adult ADHD Self-Report Scale v1.1, Barrett Impulsiveness Scale-11, adult and adolescent Autism Quotient, Kessler Psychological Distress Scales, and Disruptive Behaviour Disorder Rating Scale).

**Results:**

Individuals with XLI generally exhibited normal sensory function. Boys with XLI were at increased risk of developmental disorder, whilst adults with the condition were at increased risk of both developmental and mood disorders. Both adult and younger XLI groups scored significantly more highly than male general population norms on measures of inattention, impulsivity, autism-related traits, psychological distress and disruptive behavioural traits.

**Conclusions:**

These findings indicate that both adult and younger males with XLI exhibit personality profiles that are distinct from those of males within the general population, and suggest that individuals with XLI may be at heightened risk of psychopathology. The data are consistent with the notion that STS is important in neurodevelopment and ongoing brain function, and with previous work suggesting high rates of developmental disorders in boys with XLI. Our results suggest that individuals with XLI may require medical care from multidisciplinary teams, and should help to inform genetic counselling for the condition.

## Introduction

X-linked ichthyosis (XLI) is a dermatological condition characterised by large, dark brown scales occurring primarily on the extensor surfaces and trunk [1]. Most cases of XLI are caused by an X-linked genetic mutation resulting in deficiency for the enzyme steroid sulfatase (STS), an enzyme which cleaves sulfate groups from a range of steroids; the accumulation of cholesterol sulfate in the stratum corneum as a consequence of STS deficiency in XLI results in increased intracellular stability and cohesion, and, ultimately, partial retention hyperkeratosis and scaling [1]. 80–90% of XLI cases are caused by complete or partial deletion of the *STS* gene, with the remaining cases being attributed to point mutations within the gene. The typical deletion encompasses *STS* and a small number of adjacent genes (including members of the *VCX* family, *HDHD1A*, and *PLPNA4*) whilst larger deletions can encompass the *KAL* and *NLGN4X* genes [2]. Diagnosis of the condition is typically made on the basis of skin appearance and family history, with additional confirmatory biochemical and/or genetic analyses performed for some individuals [1].

As it arises from an X-linked mutation, XLI almost exclusively affects males, although carrier females may exhibit mild associated phenotypes such as dry skin. The XLI prevalence rate, based upon clinical diagnoses, is ~1 in 6000 males [1]; however, population-based endocrinological and genetic screening suggests that up to ~1 in 1500 males may be affected by steroid sulfatase deficiency (equivalent to ~2.5million males worldwide) indicating that lack of the STS enzyme is associated with a spectrum of phenotypic severity [3,4]. Individuals with XLI may present with a range of other extracutaneous features including: corneal opacities that do not usually affect visual acuity (10–50% of cases), cryptorchidism and testicular germ cell cancer (~20% of cases) and male pattern baldness [1]. Larger gene deletions can be associated with symptoms of contiguous gene syndromes including epilepsy, mental retardation, skeletal and craniofacial abnormalities, hypogonadotropic hypogonadism and anosmia [1].

In terms of behavioural features, there is some limited evidence from case studies [5–7], and a more systematic study in 25 boys with XLI [2], that males with STS deficiency may be at increased risk of developing Attention Deficit Hyperactivity Disorder (ADHD)(notably the inattentive subtype), and that individuals with larger genetic deletions including *NLGN4X* may be particularly susceptible to autism spectrum, or related, disorders; these data are consistent with the observations that genetic variation within *STS* is associated with inattention in boys diagnosed with ADHD [8,9], and with the expression pattern of the *STS* gene in developing brain regions underlying attention and executive function [8].

Adult male mice lacking the *Sts* gene demonstrate attentional impairments relative to wildtype controls [10] with preserved, or even enhanced, motor response control [11]; adult *Sts*-deficient mice also exhibit evidence for hyperactivity, increased anxiety, and heightened levels of aggression and behavioural perseveration [12–14].

Given our lack of knowledge regarding the range of behavioural phenotypes associated with XLI, particularly in adults, we aimed to survey a comparatively-large worldwide sample of affected males and their non-affected brothers using well-validated and detailed behavioural questionnaire measures. This analysis would enable us to test the robustness, generalisability and cross-species applicability of existing limited clinical and animal model data. Our *a priori* hypotheses were that, relative to males without XLI, males with the condition would exhibit: evidence for impaired attention (but normal, or reduced, motor impulsivity), hyperactivity, increased levels of anxiety, perseveration and aggression, and a greater likelihood of having been diagnosed with one or more developmental disorders. We successfully recruited, and analysed survey data from, a large cohort of adult males and boys with XLI, and showed a number of behavioural differences compared to normative male samples which were consistent with the aforementioned hypotheses.

## Methods

### Ethics

The study was approved by Cardiff University School of Psychology Research Ethics Committee.

### Survey structure

Three slightly-different online versions of the survey were generated using Qualtrics software for completion by adult males (>18yrs) with XLI, by non-affected adult males, or by the parents of affected and non-affected boys (5–18yrs old inclusive). After an online consent form, the first part of the survey comprised demographic information including participant age, ethnic origin, country of residence, handedness and highest educational level attained. The second part of the survey asked about the basis on which the XLI was diagnosed and confirmed, and, if a genetic test was performed, the nature of the mutation. The third part of the survey asked about the participant’s sensory acuity (scored on a Likert scale from 1 = excellent to 5 = very poor for each sense), general personality type based upon the ‘Four Temperaments’ [15], any talents or disabilities of special note, and any previous diagnosis of developmental, psychiatric or neurodegenerative illness.

Participants were then asked to complete a series of five psychological questionnaires selected on the basis of previous data: i) the Adult ADHD Self-Report Scale version 1.1 (ASRS v1.1), a five-point response (rarely-very often, 0–4) 18-item Likert-scale screening questionnaire assessing recent attentional (nine items), and hyperactive-impulsive (nine items) traits based on diagnostic criteria for ADHD and previously established for use in adults [16] and adolescents [17]; the presence of four or more symptoms (indexed by scores of ≥2 or ≥3 depending on the question) from Part A is consistent with, but not diagnostic for, a presentation of ADHD ii) the Barrett Impulsiveness Scale-11 (BIS-11), a four-point response 30-item Likert-scale questionnaire assessing aspects of impulsivity [18] for which total scores, second-order factor scores (attentional, motor and planning impulsivity) and first-order factor scores (attention, cognitive instability, motor performance, perseverance, self-control and cognitive complexity) were calculated, iii) the adult [19] or adolescent [20] version of the Autism Quotient (AQ), an extensively-validated four-point response 50-item Likert-scale questionnaire assessing behavioural traits associated with autism spectrum disorders; total and sub-scale scores (social skill, attention-switching, attention to detail, communication and imagination) were calculated, iv) the Kessler Psychological Distress Scale [21], a five-point response 10-item (K10, used for adults) or 6-item (K6, used for younger males) Likert-scale questionnaire assessing recent depressive or anxiety-related traits. K10 items were scored from 1–5 (total score range 10–50) with a score ≥20 being consistent with significant psychological distress, whilst K6 items were scored from 0–4 (total score range 0–24) with a score of ≥13 being regarded as suggestive of psychological distress and v) the Disruptive Behaviour Disorder Rating Scale (DRD-RS), a four-point response (0–3) 45-item (41 items used) Likert-scale questionnaire assessing attentional, impulsive, confrontational, disruptive and aggressive symptoms [22]; subscale scores were calculated for inattentive, hyperactive-impulsive, oppositional defiant disorder (ODD) and conduct disorder (CD) related traits.

The survey concluded with a short debrief.

### Participant recruitment and survey completion

The study and the survey URL were advertised worldwide via ichthyosis support groups and their websites (in some cases after additional ethical and academic review), social media sites (including relevant Facebook pages, and Twitter), and through dermatology departments. Participants completed the survey anonymously, either on behalf of themselves (affected or non-affected adults), or their sons (affected or non-affected individuals ≤18yrs of age), and results were returned to the research team upon completion. Data were collected between 15^th^ December 2015 and 1^st^ June 2016.

### Data analysis and statistics

Questionnaires were analysed as previously described in the literature. In the rare circumstance of participants not answering specific questions (which occurred <2.5% of the time), mean values for the experimental group were imputed. Summary data are reported as percentages, or mean values ± standard error of the mean. Categorical data were analysed using Chi-Squared with Yates’ correction. Normality of data was assessed using Shapiro-Wilk test. Testing to compare behavioural measures in the XLI sample to the ‘average’ or mean score was done using a One Sample t-test or Wilcoxon Signed Ranks Test. Traits within the same set of individuals were compared using paired t-test or Related Samples Wilcoxon Signed Rank test. Data from individuals with XLI and normative control samples were compared using Welch’s t-test with the degrees of freedom calculated using the Welch-Satterthwaite equation. P-values were calculated from the resultant t-statistic and degrees of freedom using GraphPad software. P-values <0.05 were regarded as nominally-significant.

### Data availability

Raw survey data are available in the Supporting Information (S1 and S2 Tables).

## Results

### 1. Responses and participant demographic and diagnostic characteristics

We obtained self-report data on a total sample of 58 adult males with XLI, and parent-report data on a total of 24 boys with XLI; the demographics and diagnostic information associated with these two samples are summarised in **Table 1**. Despite our efforts to recruit an optimal control group, we only received survey data from two adult non-affected brothers and one younger non-affected male. As these low numbers precluded meaningful statistical comparison with the XLI groups for subsequent analyses we compared the XLI group data to the best-available age, gender and ethnicity/culturally-matched normative data.

**Table 1:** Demographic and diagnostic information for adult and younger male XLI samples.

		Adults with XLI (n = 58)	Younger males with XLI (n = 24)
**Age (yrs)**		42±2	11±1
**Country of residence**	**United Kingdom (UK)**	23	7
	**United States of America**	19	14
	**Europe (excluding UK)**	6	0
	**Canada**	4	0
	**Rest of the world**	6	3
**% Right-handed**		84	63
**Ethnicity**	**White European or Hispanic**	46	20
	**African or Afro-Caribbean**	1	1
	**Asian**	7	0
	**Other or no response**	4	3
**Highest level of education**	**No formal education**	3	N/A
	**High school**	21	
	**College or University**	32	
	**Postgraduate**	2	
**Basis of XLI diagnosis**	**Skin appearance and family history alone**	45	12
	**Skin appearance, family history and biochemical test**	1	1
	**Skin appearance, family history and genetic test**	5	10
	**Don’t know or no response**	7	1
**Test result for individuals undergoing genetic analysis**	**Typical deletion**	1	2
	**Atypical (large) deletion**	1	1
	**Mutation specific to STS**	0	3
	**Don’t know or no response**	3	4

### 2. Sensory function

Sensory function across all five domains for the adult XLI sample (n = 57) was reported as being significantly better than average (p<0.001). Low numbers of individuals reported ‘very poor’ sensory function (vision: 1/57, hearing: 2/57, taste: 1/57, smell: 3/57 and touch: 1/57).

Sensory function across all five domains was parent-reported as being significantly better than average (score of 3) for the 24 boys with XLI (p<0.05 for vision, p<0.005 for hearing and touch, p<0.001 for taste and smell); no boys with XLI were reported to have significant sensory impairments (parental ratings of ‘very poor’).

### 3. Personality types and talents

Within the adult XLI sample, participants self-identified most closely with the following personality attributes: ‘optimistic and social’ (12/56, 21%), ‘short-tempered and irritable’ (16/56, 29%), ‘analytical and quiet’ (21/56, 37%) and ‘relaxed and peaceful’ (7/56, 13%). Comparing these frequencies to large-scale worldwide gender-specific data from a commercial company assessing personality traits [23] indicates that whilst the proportion of individuals exhibiting ‘analytical and quiet’ or ‘relaxed and peaceful’ type temperaments is relatively consistent across our XLI sample and the general male population (37% vs. 37.5% and 13% vs. 18% respectively), there is a comparatively low proportion of individuals within the XLI sample who regard themselves as ‘optimistic and social’ (21% vs. 34% in general male population), and an elevated proportion who regard themselves as ‘short-tempered and irritable’ (29% vs. 10.5% in general male population). Nine individuals within the adult XLI sample (16%) reported significant creative or artistic talents, whilst one reported notable athletic talent.

Consistent with the adult personality data, a relatively high proportion of boys with XLI were reported as being ‘short-tempered and irritable’ (9/23, 39%); the remainder were classified as being ‘optimistic and social’ (35%), ‘analytical and quiet’ (17%) or ‘relaxed and peaceful’ (9%). Talents of note within the younger XLI sample included impressive memory (n = 2), reading (n = 1), athletic (n = 1) or mathematical skills (n = 1).

### 4. Previous neurodevelopmental, psychiatric or neurodegenerative diagnoses

A high proportion of the adult male XLI sample (18/54, 33%) reported a previous diagnosis of a mental health disorder: 11/54 (20%) individuals reported a previous mood disorder diagnosis, 6/54 (11%) individuals reported a previous developmental disorder diagnosis, and one individual did not provide any further information (**Table 2**). Mood disorders were diagnosed at 23±4yrs, and developmental conditions at 11±2yrs.

**Table 2:** Existing diagnoses of evelopmental, psychiatric or neurodegenerative disorders for adult and younger male XLI samples.

		Adults with XLI (n = 54)	Younger males with XLI (n = 22)
**Individuals with a previous diagnosis of mental health disorder**		18	13
**Mood disorder**	**Depression alone, or with comorbid anxiety or OCD**	9	0
	**Bipolar disorder alone, or with comorbid ADHD or OCD**	2	1
**Developmental disorder**	**Autism spectrum disorder alone, or with comorbid dyslexia or panic disorder**	3	0
	**Autism spectrum disorder with comorbid ADHD or Irlen syndrome**	0	4
	**Sensory processing disorder**	0	1
	**Delayed speech and articulation**	0	1
	**Severe dyslexia**	0	1
	**Learning disability**	1	0
	**Migraine**	1	0
	**Unspecified disorder with features of schizophrenia and ADHD**	1	0
	**ADHD/ADD alone**	0	4
**No further diagnostic information provided**		1	1

A high proportion of boys with XLI (13/22, 59%) were reported to have had a previous diagnosis of a mental health disorder: 11/22 (50%) boys had been diagnosed with a developmental disorder, 1/22 (5%) had been diagnosed with a mood disorder, and no specific diagnostic information was provided for the remaining individual (**Table 2**). Developmental disorders were diagnosed at 6±1yrs, and the boy with a mood disorder was diagnosed at 5yrs. The high rate of developmental disorders in boys with XLI was maintained (7/17 boys, 41%) even after data from UK subjects that could potentially have been included in [2] were removed.

### 5. Adult ADHD Self-Report Scale (ASRS)

A high proportion of adult males with XLI (22/49, 45%) met the criteria for further investigations for possible ADHD i.e. ≥4 symptoms in Part A of the screening questionnaire (**Table 3**). Of the adult XLI individuals with a prior psychiatric diagnosis 12/17 (71%) had a symptom count of four or greater in Part A of the ASRS. The total inattentive symptom count was significantly higher than the total hyperactive-impulsive symptom count (p = 0.003); total inattention scores were also significantly higher than total hyperactive-impulsive scores (p = 0.003).

**Table 3:** Symptoms and scores from the Adult ADHD Self-Report Scale for adult and younger male XLI samples.

	Adults with XLI (n = 49)	Younger males with XLI (n = 19)
**Individuals with ≥4 symptoms in Part A**	22 (45%)	15 (79%)
**Total symptoms**	9 (95%CI: 5–11)	15 (95%CI: 9–16)
**Inattentive symptoms**	5 (95%CI: 3–6)	8 (95%CI: 7–9)
**Hyperactive-impulsive symptoms**	2 (95%CI: 2–4)	7 (95%CI: 3–7)
**Total score (0–4 for all 18 items)**	36.7±1.7	47.3±3.5
**Score (0–4 for 6 Part A items)**	12.8±0.6	16.4±1.3
**Score (0–4 for 12 Part B items)**	23.9±1.2	31.0±2.3
**Score (inattention items)**	20 (95%CI: 17–22)	25.1±1.7
**Score (hyperactive-impulsive items)**	15 (95%CI: 13–18)	22.2±2.0

A very high proportion of younger males with XLI (15/19, 79%) met the criteria for further investigations for possible ADHD (**Table 3**), including 6/6 boys previously diagnosed with ADHD and three boys reported to have mutations specific to *STS*. As in the adult males with XLI, there was a tendency for inattentive symptoms and scores to be higher than hyperactive-impulsive symptoms and scores (symptoms: p = 0.06, and scores: t[18] = 2.02, p = 0.06)(**Table 3**). The three boys with *STS* mutations exhibited scores consistent with the XLI sample overall (total score: 41.3±9.8, total symptom count: 11.7±3.2). As the ASRS is primarily used as a screening tool, there is currently little age and gender-matched normative data available in the literature.

### 6. Barratt Impulsiveness Scale (BIS-11)

BIS-11 data from the adult XLI group (n = 47) were compared to a normative sample of 393 male college student and community-recruited participants (21.3±0.13yrs) designed to be reflective of the general population of the USA [24]. For most measures, including total score, adult males with XLI demonstrated significantly higher scores than healthy males from the normative group (**Table 4**); the mean total score for the adult XLI group was towards the high end of the normal range for impulsiveness (52–71) [24]. However, adult males with XLI did not differ from healthy adult males on the second-order factor of motor impulsivity, and the contributory first-order ‘motor’ aspect of this (indeed, for the ‘motor’ measure, males with XLI exhibited lower scores on average). There was also no significant difference between the two groups with respect to the first-order factor of ‘cognitive instability’, although there was a strong trend for higher scores in the XLI group (p = 0.06).

**Table 4:** Scores on the Barratt Impulsiveness Scale Version 11 (BIS-11) in adult and younger male XLI samples, and normative samples.

	Adult males with XLI (n = 47)	Normative adult male sample (n = 393, [24])	Statistical comparison	Younger males with XLI (n = 17)	Normative adult male sample (n = 147, [25])	Statistical comparison
**Total score**	68.8±2.8	62.8±0.5	t[50] = 2.86, p<0.01	81.8±4.1	66.0±0.7	t[17] = 3.85, p<0.005
**Attentional impulsivity**	18.9±0.7	16.8±0.2	t[53] = 2.87, p<0.01	21.3±1.5	17.4±0.3	t[17] = 2.60, p<0.05
**Motor impulsivity**	23.6±0.7	22.4±0.2	t[51] = 1.57, p = 0.12	26.7±1.2	21.2±0.3	t[17] = 4.44, p<0.0005
**Non-planning impulsivity**	26.3±1.0	23.6±0.2	t[50] = 2.68, p<0.01	33.7±1.6	27.5±0.4	t[17] = 3.83, p<0.005
**Attention**	11.8±0.5	10.3±0.1	t[54] = 2.95, p<0.005	14.3±1.1	-	-
**Cognitive instability**	7.1±0.3	6.4±0.1	t[52] = 1.95, p = 0.06	8 (95%CI: 6–9)	-	-
**Motor**	15.0±0.6	15.2±0.1	t[51] = -0.26, p = 0.80	17.6±0.9	-	-
**Perseverance**	8 (95%CI: 8–10)	7.2±0.1	t[54] = 4.11, p = 0.0001	9.1±0.5	-	-
**Self-control**	13.8±0.6	12.4±0.2	t[51] = 2.14, p<0.05	19.3±0.9	-	-
**Cognitive complexity**	12.5±0.5	11.3±0.1	t[51] = 2.40, p = 0.02	14.5±0.8	-	-

BIS-11 scores from boys with XLI (n = 17) were compared to a normative sample drawn from secondary schools in northern Italy, and comprising 140 boys (mean age ~15yrs) [25]; only self-reported total and second-order scores were available for the normative sample. For all four of these measures, boys with XLI scored significantly more highly than boys from the normative sample (**Table 4**). Boys who had previously been diagnosed with ADHD scored particularly highly on the BIS-11 (total score 94.8±3.1) confirming its sensitivity to impulsiveness. The three boys with mutations specific to *STS* scored comparably to the rest of the XLI sample (total score: 74.0±8.4).

### 7. Autism Quotient (AQ)

Data from adult males with XLI (n = 45) were compared to two normative samples: i) 76 adult males (mean age ~37yrs) drawn randomly from the population of East Anglia, UK in which total AQ score and subscale measures were reported [19] and ii) 152,310 adult males (37.7±0.04yrs) from the general UK population recruited via a television programme in which only the total AQ score was reported [26]. Adult males with XLI scored significantly more highly (i.e. a greater number of autism-related traits) than both normative samples on the total AQ score measure; the former group also scored significantly more highly than controls on subscale measures of social skill, attention-switching, communication and imagination, but not on the ‘attention to detail’ subscale (**Table 5**).

**Table 5:** Scores on the Autism Quotient (AQ) in adult and younger male XLI samples, and normative samples.

	Adult males with XLI (n = 45)	Normative adult male sample(n = 76 [19]^a^; n = 152,310 [26]^b^)	Statistical comparison	Younger males with XLI (n = 17)	Normative younger male sample (n = 25, [20])	Statistical comparison
**Total score**	23.9±0.9	17.8±0.3^a^	t[103] = 5.16, p<0.0001^a^	27 (95%CI: 16–32)	20.2±1.0	t[20] = 2.33, p<0.05
		21.6±0.02^b^	t[44] = 2.63, p = 0.01^b^			
**Social skill**	4.7±0.3	2.8±0.3	t[97] = 4.31, p<0.0001^a^	3 (95%CI: 1–7)	2.2±0.4	t[23] = 2.41, p<0.05
**Attention switching**	6.1±0.3	4.3±0.2	t[90] = 4.92, p<0.0001^a^	6.6±0.4	5.0±0.3	t[34] = 2.89, p<0.01
**Attention to detail**	4.7±0.3	5.2±0.3	t[102] = -1.23, p = 0.22^a^	5.6±0.5	5.8±0.5	t[38] = -0.27, p = 0.79
**Communication**	4.1±0.3	2.8±0.2	t[96] = 3.47, p<0.001^a^	5.7±0.8	2.9±0.4	t[23] = 3.34, p<0.005
**Imagination**	4.3±0.3	2.7±0.2	t[88] = 4.25, p<0.0001^a^	4.3±0.7	4.4±0.4	t[28] = -0.06, p = 0.95

Data from the younger male XLI sample (n = 17) were compared to a normative sample comprising 25 boys (~13.6yrs) drawn from mainstream schools in East Anglia, UK in which parental ratings were used to derive total AQ and subscale scores [20]. Boys with XLI scored significantly more highly than control boys with respect to total AQ score; consistent with the pattern seen in the adult sample, boys with XLI scored significantly more highly than boys from the general population on measures of social skill, attention switching and communication, but scored equivalently on the ‘attention to detail’ subscale (**Table 5**). Boys with XLI also performed no differently to boys from the general UK population with regards to a measure of imagination. The boys with XLI who had previously been diagnosed with an autism spectrum condition exhibited high overall scores on the Adolescent AQ (41±0.5), confirming the sensitivity of this instrument to autism-related traits. The three boys reported to have specific mutations within *STS* scored, on average, lower than the mean for the XLI group with respect to overall AQ score (17.6±2.8 vs. 26.5), although this was not significant (t[2] = -3.23, p = 0.08).

### 8. Kessler Psychological Distress Scale

Data from the adult XLI sample (n = 44) were compared to normative data from a community-recruited Australian adult male sample comprising 756 individuals (35–44yrs) [27]. Total scores in the XLI sample were approximately double those seen in the normative sample (28.4±1.4 vs. 14.5±0.3 respectively, t[46] = 9.50, p<0.0001), and were in the range consistent with the presence of a moderate mental disorder [25–29]. As expected, the 11 adult males with XLI who had previously been diagnosed with depression, bipolar disorder or an anxiety disorder scored particularly highly on the K10 measure (34.1±2.8).

Data from the younger male XLI sample (n = 17) were compared to normative data from a large sample of male schoolchildren (n = 52,847) recruited from Kentucky, USA [28]. In our sample, 3/17 (18%) boys met the criterion for possible psychological distress (score of ≥13), two of whom had previously been diagnosed with an autism spectrum condition (one with comorbid ADHD and ODD). In the normative sample 10.2% of boys met the criterion for possible psychological distress; the frequencies across the XLI and normative groups were comparable (χ^2^_1_ = 0.41, p = 0.52). The mean total score for the XLI sample was 7.1±1.3; the three boys with mutations specific to *STS* scored lower than this (3.7±1.3), but not significantly so (t[2] = -2.61, p = 0.12).

### 9. Disruptive Behaviour Disorder Rating Scale (DBD-RS)

Adult males with XLI (n = 40) self-reported whether they currently exhibited, or had previously exhibited, a range of traits related to ADHD, Oppositional Defiant Disorder and Conduct Disorder. Five individuals (13%) met criteria consistent with an ADHD diagnosis i.e. ≥6 inattentive and/or ≥6 hyperactive-impulsive symptoms; of these, four individuals (80%) presented with features of predominantly-inattentive ADHD, and the remaining individual presented with features of predominantly-hyperactive-impulsive ADHD. Across the entire adult XLI sample, there was no significant difference between the number of inattentive symptoms and the number of hyperactive-impulsive symptoms (1 (95%CI: 1–3) vs. 2 (95%CI: 1–2) respectively, p = 0.12) but the total inattentive score was significantly higher than the total hyperactive-impulsive score (9 (95%CI: 7–10) vs. 7 (95%CI: 4–8), p = 0.047). A high proportion (14/40, 35%) of adult males with XLI exhibited symptoms consistent with a diagnosis of Oppositional Defiant Disorder (≥4 symptoms). Across the entire XLI adult sample, the total ODD item score was 8.4±0.9. Three of the individuals exhibiting symptoms consistent with ODD also exhibited symptoms consistent with conduct disorder (8% of the entire adult XLI sample). Five individuals (13% of the overall sample) admitted to ‘pretty much’ or ‘very much’ aggression towards people or animals. The DBD-RS is not typically used as a self-report measure in adults; hence, there are no appropriate normative data available.

Parents reported on whether boys with XLI (n = 17) exhibited a range of behaviours associated with ADHD, Oppositional Defiant Disorder and Conduct Disorder. Nine boys (53%) met criteria consistent with an ADHD diagnosis (of whom four had been formally diagnosed with the condition); of these nine boys, four (44%) presented with features of predominantly inattentive ADHD, and the remainder presented with features consistent with combined-presentation ADHD. Across the whole sample, there was no significant difference between the number of inattentive symptoms and the number of hyperactive-impulsive symptoms (6 (95%CI: 1–9) vs. 4 (95%CI: 0–7) respectively, p = 0.07) although the total inattentive score was significantly higher than the total hyperactive-impulsive score (15.4±1.9 vs. 12.0±2.1, t[16] = 2.35, p = 0.03). A high proportion (6/17, 35%) of boys with XLI exhibited symptoms consistent with a diagnosis of Oppositional Defiant Disorder, including 2/3 boys with a mutation within *STS*. Across the entire XLI younger male sample, the median total ODD item score was 8 (95%CI: 6–11). One individual exhibiting symptoms consistent with ODD also exhibited symptoms consistent with conduct disorder (6% of the entire younger male XLI sample). Two individuals (12% of the entire younger male XLI sample) were reported to have demonstrated ‘pretty much’ or ‘very much’ aggression towards people or animals.

The data from boys with XLI were compared to three normative samples: i) a sample of 396 boys (aged 14–18, 37% Caucasian) recruited from schools across four US states for whom total teacher-rated DBD-RS inattentive, hyperactive-impulsive and ODD scores were available [29], ii) a sample of 149 boys (aged 11–13yrs) recruited from 22 school districts across the US (and representative of the ethnicity of the US general population) for whom parent-rated total inattention and hyperactive-impulsive scores on the ADHD-RS IV (equivalent to total inattention and hyperactive-impulsive scores obtained on the DBD-RS) were available [30] and iii) a sample of 11-year old boys (n = 159–164) drawn randomly from the general school population across Sweden [31] for which mean inattention, hyperactive-impulsive, ODD and CD scores from DRD-RS were available (Prof. Ata Ghaderi pers. comm.). In comparison to the first two normative samples, boys with XLI showed evidence for substantially higher levels of inattentive and hyperactive-impulsive traits, whilst their total ODD scores on the DBD-RS were significantly higher than those reported for the normative sample in [29](**Table 6**). Comparison with the Swedish normative data also indicated significantly increased inattentive (t[17] = 6.06, p<0.0001), hyperactive-impulsive (t[16] = 4.49, p<0.0005) and ODD (t[16] = 4.48, p<0.0005) traits in the XLI sample, but not increased CD scores (t[16] = 1.56, p = 0.14).

**Table 6:** Scores on the Disruptive Behaviour Disorder Rating Scale (DBD-RS) in the younger male XLI sample, and normative samples.

	Younger males with XLI (n = 17)	Normative younger male sample (n = 396 [29]^a^; n = 149 [30]^b^)	Statistical comparison
**Total inattention item score**	15.4±1.9	8.3±0.4^a^	t[17] = 3.69, p<0.005^a^
		6.7±0.5^b^	t[18] = 4.43, p<0.0005^b^
**Total hyperactive-impulsive item score**	12.0±2.1	5.6±0.3^a^	t[16] = 3.02, p<0.01^a^
		4.8±0.5^b^	t[17] = 3.34, p<0.005^b^
**Total Oppositional-Defiant Disorder score**	8 (95%CI: 6–11)	4.5±0.3^a^	t[17] = 3.42, p<0.005^a^

## Discussion

X-linked ichthyosis (XLI) is a dermatological condition arising from a deficiency for the enzyme steroid sulfatase (STS). *STS* is highly expressed in the developing and adult brain, and can potentially influence neurodevelopment and ongoing brain function via a number of direct and indirect mechanisms [32]. Previous data have indicated increased rates of developmental disorders and associated behavioural phenotypes in individuals lacking STS.

In the present study, we successfully recruited a large male sample with XLI online and obtained detailed anonymous information on their personality and behaviour using well-validated questionnaire measures. One advantage of such an approach is that respondents are more likely to provide accurate answers to sensitive questions [33]. On the basis of these data, we are able to provide: a) a behavioural and psychiatric profile of adults with XLI for the first time, b) a detailed analysis of areas of altered psychological function in adults and younger males with XLI, and c) a replication of initial clinical data in boys with XLI. Whilst our attempts to recruit the optimal control group of non-affected brothers of individuals with XLI were unsuccessful, as our sample was broadly representative of the general populations of UK and USA, we could compare our samples to one or more appropriately age, gender, and ethnicity/culturally-matched normative samples.

Our first main finding was that sensory function was generally regarded as being average, or better-than-average, across all domains for both adult and younger male XLI samples. This is important given the fact that XLI has been associated with anosmia and corneal opacities in some cases [1], and that steroid sulfatase is expressed in the developing tongue [8]. Additionally, it suggests that sensory impairments in individuals with XLI are unlikely to explain any behavioural or psychiatric differences from unaffected males.

Consistent with the data in [2], boys with XLI exhibited higher rates of autism spectrum disorder and ADHD diagnoses than expected in boys from the general population (ASDs: 7.5% vs. 2.4% [34]; ADHD: 7.5% vs. 3–4% [35]). Interestingly, however, scores indicative of ‘psychological distress’ (K6) in the younger male XLI sample did not differ significantly from those seen in normative samples; this apparent discrepancy could be due to parental as opposed to self-reporting of symptoms, or that the disorders did not substantially impair normal functioning in these boys. In terms of AQ-indexed autism-related traits, younger males with XLI showed altered performance in the areas of social skill, communication and attention-switching (‘perseveration’), but similar levels of performance to control males with regards to imagination and ‘attention to detail’. In terms of ADHD-related traits, data from the ASRS, BIS-11 and DRD-RS provided convergent evidence that boys with XLI exhibited significantly impaired attention, and elevated levels of activity and impulsivity (across all domains), relative to boys without XLI. The DRD-RS data indicated that 53% of boys with XLI have symptoms consistent with a diagnosis of ADHD, a figure comparable to the 40% figure obtained previously on the basis of detailed clinical assessment of boys with XLI [2]. These previous clinical assessments further indicated that boys with XLI may be more susceptible to the inattentive presentation of ADHD; our data replicate this finding, in that our sample of boys are more likely to present with features of inattentive (or combined) presentation ADHD than hyperactive-impulsive presentation ADHD, and present with more inattentive than hyperactive-impulsive features. With respect to disruptive behavioural disorders, ~35% boys with XLI demonstrated behavioural features consistent with a diagnosis of ODD and ~6% demonstrated features consistent with a diagnosis of conduct disorder (6%); these estimates appear comparatively high given that the point prevalence of ODD and CD in young males is reported to be ~4.6% [36]; moreover, a relatively high proportion of boys with XLI (12%) were reported to have displayed considerable aggression towards other people or animals. However, the finding that mean CD scores on DRD-RS were not significantly from a normative sample means that, as yet, there is no strong converging evidence to support the idea that younger individuals with XLI are at increased risk of CD. A small proportion of boys with XLI may exhibit savant-like skills in the areas of memory, reading and mathematics.

For the younger male sample, genetic information was available for some individuals. Boys possessing a mutation within *STS*, or with the typical deletion, generally exhibited a behavioural profile typical of the whole group, indicating that it is the lack of STS which predisposes to most of the observed behavioural phenotypes. However, for autism-related traits, boys with *STS*-limited mutations scored comparatively low on the total AQ measure, and the boy with an atypical larger genetic deletion scored very highly (42); these data suggest the possibility that, in line with previous work, one or more genes contiguous with *STS*, most likely *NLGN4X* [2], may also contribute towards autism-related traits.

Adult males with XLI self-reported high levels of recent ‘psychological distress’ (incorporating depression and anxiety-related traits) when compared to unaffected males (K10), and an increased likelihood of presenting with a ‘short-tempered and irritable’ personality profile; consistent with this, rates of diagnosed mood and autism spectrum disorders were higher in adult males with XLI than in males from the general population (~20% vs. ~4% [37], and ~6% vs. ~1% [38] respectively).

In the adult male XLI sample, as in the younger male sample, scores on the social skill, communication and attention-switching aspects of the AQ were significantly higher than for controls; like in the younger males, the measure of ‘attention to detail’ was relatively unaffected in individuals with XLI suggesting that individuals with XLI do not exhibit the weak central coherence commonly seen in individuals with idiopathic autism [39]. In contrast to younger males, adult males with XLI showed impairments in imagination relative to controls; this phenotype may therefore develop with age in individuals with XLI. Alternatively, this apparent age effect could arise from the contrast between self-reporting of traits in adults, and parental reporting of traits for the younger sample.

Although no adults with XLI in our sample had been formally diagnosed with ADHD, screening for symptoms suggested that a disproportionately high number of them could potentially meet diagnostic criteria (13% by the DRD-RS screening criteria vs. ~2.5% in general population samples [35]). Across the entire adult XLI sample, both inattentive and hyperactive-impulsive scores were significantly higher than for comparison control samples, and, as in the younger sample, inattention seemed to be relatively more affected than hyperactivity-impulsivity. Data from the BIS-11 suggested an interesting dissociation whereby whilst most types of impulsive behaviour were greater in adult males with XLI than in adults without the condition, for motor impulsivity there was no group effect; this pattern of results resembles what we have previously seen in mice, where adult male animals lacking the *Sts* gene (or mice with the STS enzyme inhibited) exhibit normal, or even enhanced, motor impulsivity relative to controls [11]. The fact that motor impulsivity levels are significantly higher in boys with XLI than controls, but not in adults with XLI relative to controls, suggests the alternative possibilities that this phenotype manifests as a function of age, or that it is sensitive to self- versus parental reporting.

The observation that adults who had been diagnosed with a psychiatric disorder (including mood disorders) exhibited high levels of inattentive and hyperactive-impulsive traits, suggests that the genetic mutations associated with XLI may have pleiotropic effects on developmental and mood-related phenotypes, as has been documented for numerous psychiatric genetic risk loci [40]; it is plausible that high levels of childhood ADHD-related traits in individuals with XLI may predict later adult psychopathology, including mood disorders. Rates of putative ODD and CD were similarly high between adult and younger male samples. Unfortunately, the lack of genetic data on the adult male XLI sample precluded any meaningful analysis examining genotype-phenotype correlations.

Whilst this study has generated some interesting findings, there are a number of issues that should be considered in its interpretation. First, we are completely reliant upon accurate self, or parental reporting, as the information provided on an individual’s diagnoses, genetic mutation, or behavioural phenotype is not corroborated by clinical evidence or by more objective phenotyping; notably we are reliant on clinical diagnoses which worldwide have a notoriously low inter-rater reliability across some regions, and participants’ responses may have been shaped by their knowledge of previous research findings on behavioural phenotypes in XLI [2]. The absence of a diagnostic psychiatric interview means that we were unable to examine potentially confounding features on psychiatric and behavioural presentations (e.g. the extent to which symptoms were impairing, and the extent to which symptoms fluctuated as a function of an individual’s medication regime). Second, our two samples have a wide age range (5–18yrs, and 19–82yrs) and show a degree of heterogeneity in terms of country of residence, diagnostic and confirmatory procedures, and highest level of education. Third, our survey was only available to computer-literate English-speaking individuals and was most widely-advertised in countries with active support groups for ichthyosis-related conditions, raising the possibility that the findings are only relevant to specific geographical and cultural contexts. Fourth, we were also reliant upon different normative samples from around the world for each of the questionnaires, which may feasibly have introduced a degree of noise into the comparisons. There is also the possibility that the magnitude of differences between the XLI samples and the general male population may have been exaggerated by response bias, whereby individuals are more likely to respond to the survey if they, or their sons, have experienced substantial behavioural or psychiatric issues. Fifth, in order to best compare behavioural profiles in younger and older males with XLI, we used essentially the same questionnaire measures for both groups; some questionnaires have not been validated for use in one of the populations (or for self vs. parental report), and they may be suboptimal for use in one of the populations. Finally, our survey was simply descriptive and did not allow us to address the psychological mechanisms that may underlie the differential behavioural and psychiatric profiles of males with and without XLI and non-affected males e.g. the higher rate of depression in males with XLI could be due to direct effects of STS deficiency on the brain, or, alternatively, could be due to psychosocial difficulties arising from living with a potentially stigmatising dermatological condition. To dissociate these possibilities, it would be interesting to examine the behavioural profile of individuals with STS deficiency, but no, or very mild, dermatological abnormalities.

We argue that the potential confounds above do not materially affect the main conclusions of the study. The samples we obtained had a demographic profile largely consistent with the general population within the countries from which they were recruited, there was no evidence for an abnormally high degree of variability within the behavioural data, there was a high degree of consistency of the pattern of effects across multiple questionnaires, measures, and normative samples, and across younger and adult male samples; these patterns were also consistent with our hypotheses, and analogous to those hinted at in previous clinical data using an XLI sample not ascertained on the basis of behavioural phenotypes [2], case studies and animal work. The general pattern of effects was consistent between individuals with XLI and well-recognised behavioural difficulties (as defined by a prior psychiatric diagnosis) and those without such a diagnosis. Finally, individuals who reported previous psychiatric disorders scored highly on questionnaires assessing traits associated with those conditions.

## Conclusions

Our data, in combination with previous findings, strongly indicate that individuals with XLI are at significantly increased risk of developmental conditions (younger males and adults) and psychiatric illness (notably mood disorders) in adults. Additionally, males with XLI differ substantially from age-matched males from the general population with regard to numerous aspects of behavioural function. Using *in vivo* techniques to examine and understand the neural basis of these phenotypes will be important for understanding the role of STS in the brain, and for developing therapeutic strategies for conditions associated with its deficiency. We hope that the present information will improve awareness of potential neurodevelopmental or neuropsychiatric comorbidities amongst healthcare professionals who typically diagnose XLI (General Practitioners and dermatologists), and will facilitate multidisciplinary care of patients across specialities and services where required. Moreover, the data presented herein will provide valuable information for professionals offering genetic counselling to individuals with XLI, and to carriers of the underlying genetic mutation.

## Supporting Information

S1 Table Raw self-report survey data from adult males with XLI. (XLSX)

S2 Table Raw parental-report survey data for younger males with XLI. (XLSX)
